# Intradermal microbubbles and contrast-enhanced ultrasound (CEUS) is a feasible approach for sentinel lymph node identification in early-stage breast cancer

**DOI:** 10.1186/s12957-015-0736-x

**Published:** 2015-11-19

**Authors:** Fei Xie, Dongjie Zhang, Lin Cheng, Lei Yu, Li Yang, Fuzhong Tong, Hongjun Liu, Shu Wang, Shan Wang

**Affiliations:** Department of Breast Disease, Peking University People’s Hospital, Beijing, China; Department of Ultrasound Diagnosis, Peking University People’s Hospital, Beijing, China; Department of Gastrointestinal Surgery, Peking University People’s Hospital, Beijing, China

**Keywords:** Sentinel lymph node (SLN), Sentinel lymph node biopsy (SLNB), Microbubbles, Contrast-enhanced ultrasonography (CEUS), Blue dye, SonoVue, Breast cancer

## Abstract

**Background:**

Microbubbles and contrast-enhanced ultrasound (CEUS) is a new technique for locating sentinel lymph node (SLN). The aim of this study is to explore the feasibility of SLNs tracing by CEUS using microbubbles in breast cancer patients and the value of enhancing patterns in diagnosing lymph nodes metastases.

**Methods:**

A clinical trial was registered (trial registration: ChiCTR-DDT-13003778). One hundred and one consecutive consenting patients with breast cancer undergoing SLN biopsy were enrolled. Before the surgery, microbubble was injected periareolarly. Lymphatic drainage pathway was detected by CEUS, and guidewire was deployed to locate the SLN before the operation. Blue dye was also used to help in tracing sentinel lymph node during the operation. The identification rate and the accuracy rate were recorded. Enhancing patterns of lymph nodes were recorded and compared with the pathological diagnosis.

**Results:**

Of the 101 cases, SLNs in 99 cases were successfully identified by at least one tracer, including 98 cases identified by CEUS and 97 cases by blue dye. There was no significant difference between the two methods (*P* = 0.705). Guidewires were deployed successfully in all 98 cases, and the localized SLNs were all isolated successfully in the following operations. The status of SLNs isolated by CEUS was completely identical to that of the whole axillary lymph node while 7.1 % cases were misdiagnosed as negative by blue dye method. The sensitivity of predicting SLNs metastases by CEUS enhancing pattern was 81.8 %, the specificity was 86.2 %, and the positive and negative predictive values were 75.0 and 90.3 %, respectively.

**Conclusions:**

Microbubbles and CEUS are feasible approaches for SLN identification. The enhancing patterns on CEUS may be helpful to recognize the metastasizing SLNs. This novel method may be a promising technique for the clinical application.

## Background

Axillary lymph node status is an important prognostic factor in patients with breast cancer [1]. Sentinel lymph node (SLN) biopsy has been the standard procedure for axillary staging in early breast cancer with clinical normal nodes [2–5]. Non-sentinel lymph node (NSLN) metastasis is detected in 35–50 % of SLN positive patients [6, 7]. Although a number of studies have investigated models or nomograms for non-sentinel lymph node (NSLN) metastasis prediction, none of them can predict the probability of NSLN metastasis correctly [8–10]. Hence, if any SLN is positive, the standard procedure remains axillary lymph node dissection (ALND).

The definition of sentinel lymph nodes is the initial lymph nodes that receive lymphatic drainage from the primary tumor. Some markers are used to label the sentinel lymph nodes along the lymphatic drainage pathway. The traditional method for sentinel lymph nodes tracing is a dual-labeled technique involving blue dye and radiolabeled colloid [11]. The identification rate of the dual-labeled technique has been shown to be up to 96 %, with false negative rates between 5 and 10 % [12, 13]. Nevertheless, the half-life of the technetium-99m (^99^mTc) is up to 6 h, while molybdenum-99 (^99^Mo) decays rapidly, which may restrict the scheduling of radioisotopes production and delivering. Handling and disposal of radioisotopes are also severe challenges for hospital management. All these drawbacks have limited the widespread use of radioisotopes for SLN biopsy. Isosulfan blue, patent blue, and methylene blue are dye tracers that are frequently used. The identification rates of SLN biopsy by blue dye alone range from 66 to 94 %, with false negative rates of 0 to 12 % [14–17]. Considering the acceptable accuracy and absence of radioactive contamination, the blue dye has become the alternative standard control tracer in many studies [18, 19]. Poor tissue penetration is the obvious drawback of blue dye technique. Surgeons have to anatomize the lymphatic vessels cautiously to find the SLN during the procedure.

The constraints of the existing SLN biopsy techniques have led to the development of alternative methods. Indocyanine green fluorescence (ICG) [18–20], superparamagnetic iron oxide nanoparticles (SPIO) [21, 22], and contrast-enhanced ultrasound (CEUS) using microbubbles have been reported as new feasible alternative techniques for the SLN biopsy in breast cancer [23–26]. Sulfur hexafluoride microbubbles is the most popular ultrasound contrast agent at present, which plays a role as an inert gas in vivo. There is no evidence of metabolism of sulfur hexafluoride. Furthermore, diameters of the bubbles range from 2–10 μm (mean 2.5 μm), which are much less than those of the red blood cells (mean 7.2 μm). So the bubbles can easily pass through the blood capillary and lymphatic microvessels, even across the alveolar epithelium. Therefore, the bubbles also can be cleared up by the lung and kidney easily. On the other hand, there are no iodine and proteins in sulfur hexafluoride microbubbles, which prevents a great portion of patients from having allergy. Sulfur hexafluoride microbubbles may be a promising SLN marker considering its safety and reliability. Nevertheless, there are very few published studies of sentinel lymph node biopsy done by CEUS using microbubbles in human beings [27, 28].

The aim of this study is to validate the effectiveness of the identification of SLNs using microbubbles and CEUS in patients with early breast cancer. Also, we attempted to explore the potential value of CEUS in the pre-operative diagnosis of SLN metastases in patients with early breast cancer.

## Methods

### Patients

The study was designed as self-control and registered at Chinese Clinical Trial Registry (the registration number is ChiCTR-DDT-13003778). The study was approved by the institutional ethics committee of Peking University People’s Hospital (IRB approval number is 2012045). Informed consent was obtained from all enrolled patients (the version number is modified revision 2012.12.18). Between October 2013 and October 2014, 101 consecutive consenting patients with early-stage breast cancer who were scheduled for SLN biopsy were recruited into the study. Exclusion criteria included inflammatory breast cancer, previous surgical approach in the ipsilateral axillary region, history of sulfur hexafluoride allergy, axillary lymph node involvement proved by histology or cytology, and stage IV disease. Patients whose axillary lymph nodes were suspiciously positive accept fine-needle aspiration cytology (FNAC) before the operation. If the pathology result was negative, there would still be an indication for SLN biopsy. Patients receiving neoadjuvant therapy were not permitted. All the patients were female and treated in Peking University People’s Hospital. Their ages ranged from 22 to 82, and the median age was 54. In the 101 patients, 49 (48.5 %) had the primary breast lesion located on left side, and 52 (51.5 %) had right breast lesions. Core-needle biopsy (CNB) was performed in 24 cases to confirm the breast cancer; FNAC was performed in the remaining 77 cases. Patients diagnosed by FNAC received a frozen pathological diagnosis during the procedure. As for breast treatment, 36 patients underwent breast-conserving surgery, and 65 patients received total mastectomy (Table 1).

**Table 1:** Patients (*N* = 100) and primary tumor (*N* = 101) characteristics

Baseline characteristics	Number of patients (*N* = 100) or cases (*N* = 101) (%)
Patient characteristics	
Menopause status	
Premenopausal	39 (39 %)
Post-menopause	61 (61 %)
BMI	
<19	3 (3)
19–25	59 (59 %)
≥25	38 (38 %)
Tumor characteristics	
Orientation	
Upper outer quadrant	46 (45.5 %)
Upper inner quadrant	27 (26.7 %)
Lower outer quadrant	10 (9.9 %)
Lower inner quadrant	9 (8.9 %)
Central quadrant	9 (8.9 %)
Tumor size	
Tis	4 (4.0 %)
T1	77 (76.2 %)
T2	20 (19.8 %)
Histology	
DCIS	4 (4.0 %)
Invasive ductal	81 (80.2 %)
Invasive lobular	8 (7.9 %)
Others	8 (7.9 %)
Stage^a^	
0	4 (4.0 %)
I	49 (48.5 %)
II	35 (34.7)
III	13 (12.9 %)
Molecular subtype [35]	
Luminal A	26 (25.7 %)
Luminal B	53 (52.5 %)
Her-2 positive	4 (4.0 %)
Triple negative	18 (17.8 %)

*DCIS* ductal carcinoma in situ

^a^According to the American Joint Committee on Cancer (AJCC) TNM staging system for breast cancer, 7th edition

### Instruments and materials

All sonographic examinations were performed by two experienced sonographer using two uniform GE Logiq E9 scanners (GE Healthcare) equipped with high-frequency linear array probes (ML6–15-D) and contrast pulse sequences (CPS). Microbubbles composed of phospholipid-stabilized membrane and sulfur hexafluoride gas in the inside (SonoVue, Bracco Imaging) were used as ultrasound contrast agent. SonoVue presented as a dry powder and needed to be prepared by 5 mL of normal saline into 8 μl/ml sulfur hexafluoride microbubbles. The ampoule was shaken vigorously more than 30 s every time before injection to ensure a homogeneous suspension of bubbles. Methylene blue (Jumpcan Pharmaceuticals) was used as a blue dye. A 7-cm-long double hook guidewire (20G, LW0077, BARD) was used to locate the sentinel lymph nodes.

### CEUS procedure

Before the SLN biopsy, patients received a gray-scale ultrasound examination of the axilla first, so the sonographers had a general idea of the position of lymph nodes. Then 1.5-mL microbubble suspension was intradermally injected into the upper lateral areola. If an incision biopsy was performed in upper outer quadrant previously, we chose the outer edge of the incision as the injection site instead. After injection, subcutaneous lymphatic drainage pathway directing to the axilla could be detected immediately on contrast pulse sequencing in most cases. Moving the probe along the lymphatic channels, the areas of contrast agent accumulation could be detected, which were the enhanced lymph nodes. Usually, the passing time from injection to axillary node mapping was between 5 and 67 s. Contrast agent remained in the SLN for up to 4 min. Massaging the areola for 10 s intensified the image again. If the lymphatic vessel was not detected successfully in a few cases, another injection was given. If no obvious lymphatic filling was obtained or no lymph node developed after two consecutive injections, the case was recorded as a failure. The conventional gray-scale window was alive synchronously to confirm the presence of an architecturally defined SLN. In some cases, an apparently enhanced lymph node supposed to be an SLN on contrast pulse sequencing was difficult to recognize in a gray-scale pattern.

Once identified, contrast-enhanced ultrasound-identified SLNs were localized with double hook guidewire. After insertion, the tails of the guidewire were covered with a sterile dressing. Considering the unavoidable frequent movements of an upper limb, and the discomfort caused by a foreign matter in the axilla, it was more appropriate to perform this procedure just before the SLN biopsy.

### SLN biopsy

Immediately after the induction of general anesthesia, 1-mL blue dye was intradermally injected into the periareolar upper outer quadrant region. If an incision biopsy was performed in upper outer quadrant previously, the outer edge of the incision was taken to be the alternative injection site. Five minutes later, standard SLN biopsies were performed by four well-trained surgeons, respectively. All blue dye-containing lymph nodes, guidewire-directing lymph nodes, and any suspicious lymph nodes during the exploration were excised and numbered in accordance with the following instructions: both blue dye- and guidewire-containing nodes (bub+/dye+) were labeled “1”; only blue dye-containing nodes were labeled (bub-/dye+) “2”, “3” for only guidewire-containing nodes (bub+/dye-), and “4” for neither blue dye- nor guidewire-containing nodes (bub-/dye-). All the lymph nodes were sent for histologic analysis. Standard axillary lymph node dissection was performed if any of the nodes proved positive.

### Statistics

The identification rate was defined by the proportion of patients with SLNs identified with either method. Data were subjected to *χ*^2^ test or Fisher’s exact test using SPSS statistical software version 17.0 (SPSS Inc. Chicago, IL, USA). The level of significance was set at *P* < 0.05.

## Results

### CEUS technical success

**Fig. 1:**
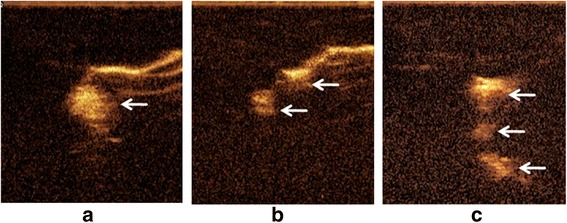
Enhanced lymph nodes (*single arrows*) on contrast-enhanced ultrasound. **a** Only one lymph node enhanced. **b** Two sequentially visualized SLNs linked by lymph vessel. **c** Three ordinally enhancing SLNs in a row

**Fig. 2:**
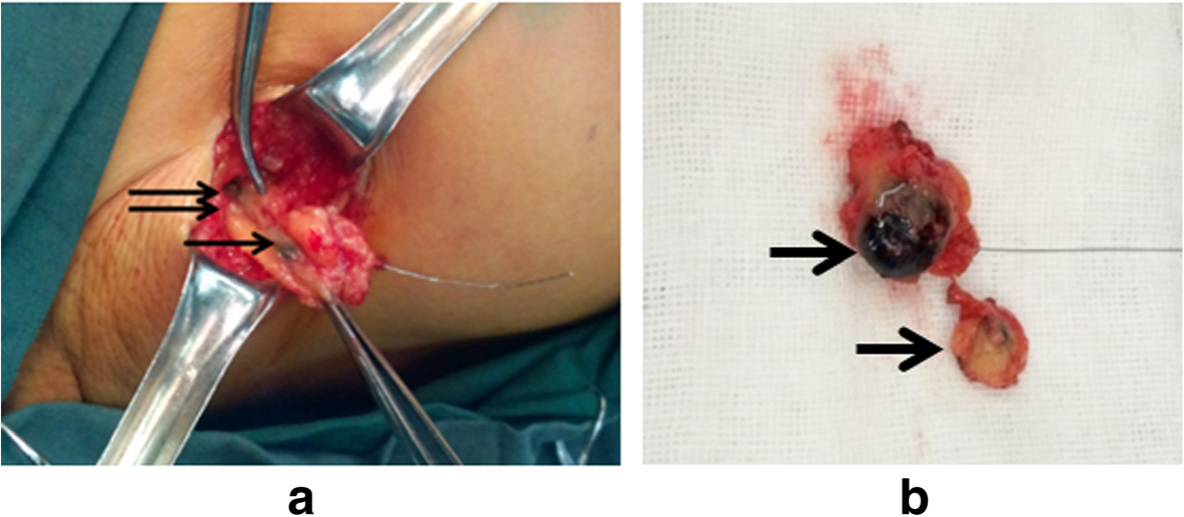
Guidewire-guided SLNs identification. **a** Excising guidewire-containing SLN (single arrow) along the wire. Another blue dye-containing lymph node (*double arrow*) was also isolated. **b** Two SLNs were excised (*single arrow*). The guidewire-containing SLN was also reported as “blue” during the procedure

In 98 of 101 cases, lymphatic channels draining from the primary lesion and at least one lymph node were clearly visualized. In the remaining 3 cases, lymphatic vessels were visualized clearly but no lymph node was visualized. These 3 cases had stage III disease, and tumor thrombus in capillaries was proved by pathological diagnoses. In the 98 lymph nodes visible cases, only one SLN was detected on CEUS in 84 cases (85.7 %), 11 cases (11.2 %) had two sequentially visualized SLNs, and 3 cases (3.0 %) had three ordinally enhancing SLNs (Fig. 1). The first one or two enhanced nodes were deployed guidewire, respectively. The procedure was successful in all the 98 cases, and SLNs marked by guidewires were isolated correctly in the following operations (Fig. 2). There was no displacement of wires, hematoma, or infections. It was worth noting that it is better to perform the insertion just before the surgery.

### SLN detection

Of the 101 cases, SLNs in 99 cases were successfully identified by at least one tracer, including 98 cases identified by CEUS and 97 cases by blue dye. The identification rates of CEUS, blue dye, and the combined method were 97.03 % (98/101), 96.04 % (97/101), and 98.02 % (99/101), respectively (*P* = 0.705). In total, 271 SLNs were excised. CEUS method detected less SLNs than blue dye method (115 vs. 211). While the positive rates of SLNs identified by CEUS was 35.7 % (41/115), significantly higher than that by blue dye (21.8 %, 46/211) (*P* = 0.001) (Table 2).

**Table 2:** Classification of SLN in terms of CEUS and blue dye

Characteristic	Number of cases (%)	Number of SLNs identified (%)	Number of positive SLNs
CEUS^+^/blue dye^+^	96 (95.0 %)	101 (37.3 %)	40
CEUS^+^/blue dye^−^	2 (2.0 %)	14 (5.2 %)	1
CEUS^−^/blue dye^+^	1 (1.0 %)	107 (39.5 %)	6
CEUS^−^/blue dye^−^	2 (2.0 %)	49 (18.1 %)	0
Total	101 (100 %)	271 (100 %)	47

Axillary lymph node involvement was confirmed in 36 cases by paraffin pathology while ALND was performed in 41 cases. The other 5 cases received ALND because suspicious positive lymph nodes were found during the sentinel lymph node biopsy (SLNB), although the frozen pathology was negative. Among the 36 axillary involved cases, SLNs in 33 cases were successfully identified by CEUS and all were diagnosed positive by frozen pathology; the other 3 cases had stage III disease and tumor thrombus in the capillaries so the CEUS method was a failure; SLNs in 34 cases were successfully identified by blue dye, but 1 case was misdiagnosed as negative (the positive SLN was labeled CEUS^+^/blue dye^−^). Furthermore, SLN identification by CEUS was independent of disease stage and molecular subtype (Table 3).

**Table 3:** SLN detection rate according to stage and subtype using the CEUS method

	Clinic characteristic	CEUS		*P*
		Detection cases	Detection rate (%)	
Stage				
0–II	88	88	100	<0.001
Stage III	13	10	76.9	
Subtype				
Triple negative	18	16	88.9	0.046
Others	93	92	98.9	

### Pattern of CEUS enhancement

**Fig. 3:**
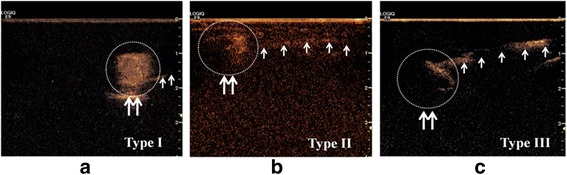
Different types of enhancing patterns; *double arrows* and *circles* direct lymph nodes; *single arrows* direct lymph vessels. **a** Type I enhancement; SLN was obviously and homogeneously enhanced. **b** Type II enhancement; the enhancement was obvious not homogeneous. **c** Type III enhancement; SLN was hardly enhanced

The pattern of enhancement of SLNs was classed into three types: type I, SLN was obviously and homogeneously enhanced; type II, SLN was obviously enhanced, but the enhancement was not homogeneous, with hypoperfusion or non-perfusion area present; type III, SLN was weakly enhanced or not enhanced (Fig 3). Comparing the enhancement pattern with paraffin pathological diagnosis of SLNs, we found that type I was the most typical pattern in non-involved SLNs (56/65), while type II enhancement was more common in involved SLNs (23/33) (*P* < 0.001). If we considered the SLNs with type II or III enhancement as positive nodes and type I as negative, the sensitivity of CEUS in the diagnosis of SLNs metastases was 81.8 % (27/33), the specificity was 86.2 % (56/65), and accuracy rate was 84.7 % (83/98). The positive predictive value was 75.0 % (27/36); the negative predictive value was 90.3 % (56/62) (Table 4).

**Table 4:** Patterns of enhancement and SLNs status

		CEUS		*P*
SLN	Type I	Type II	Type III	
Pos	6	23	4	<0.001
Neg	56	7	2	

## Discussion

SLNB has become the routine procedure in patients with early breast cancer, and ALND is still a standard treatment for SLN involved case. Although according to the ACOSOG Z0011 and IBCSG 23-01 data, patients with limited SLN metastatic breast cancer treated with breast conservation and systemic therapy may omit ALND [29, 30]. The conclusions still need to be validated by more well-designed trials. So the primary value of SLNB remains consisting in indicating axillary status with minimal trauma. The combination of radioisotopes and blue dye is a popular method for SLN tracing. However, the limitations are not negligible. The diameter of radioactive colloid is usually between 50 and 100 nm, while the endothelial gap of subcutaneous lymph vessel is between 120 and 500 nm; so the radioactive colloid cannot pass through the lymph–vessel freely but can be absorbed by endocytosis. Therefore, many hours are needed to detect SLNs after radioactive colloid is injected. Moreover, radioactive materials handling is another challenge for hospital management. A blue dye may lead to staining of adjacent adipose tissue. In addition, breast tattooing is another problem that limits its application. Anaphylaxis following blue dye injection is also an infrequent but serious risk for patients. In this study, we chose sulfur hexafluoride microbubbles as a contrast agent, which is much smaller than red blood cell; so the agent can readily cross into the SLNs and be easily eliminated. The detecting instrument is a normal ultrasound apparatus with contrast pulse sequences, and it is readily available. Moreover, there are no iodine and proteins in sulfur hexafluoride microbubbles, which prevents patients from allergy. In consideration of the receivable accuracy rate of blue dye guided method [14–17], and its convenience and fast transit, we chose blue dye as the reference substance for SLNB.

The potential application of CEUS and microbubbles in identifying SLNs in a swine melanoma model was first reported in 2004 [31]. Then, a few studies of SLN identification by CEUS and microbubbles in human beings were published. A UK study with 54 patients found 89 % identification rate by CEUS and intradermal microbubbles [24]. This result was repeated in a further 80-patient UK study [25]. In 2013, a larger study of 347 patients also found a low sensitivity of 61 % but a specificity of 100 % [26]. In the above three studies, there was no significant difference between the identification rates by CEUS and standard dual technique. However, another Japan study with 20 patients had a detecting rate of 70 %, much lower than that of γ-probe-guided method of 100 % [32]. Dual-labeled technique was used as the control method in all the above studies, but the patient numbers varied widely and there was also difference in the effectiveness. In addition, there are few reports on the relationship between the pattern of enhancement and lymph node status.

SLNB using blue dye and radioisotope often results in the removal of multiple SLNs. In this study, the identification rate of CEUS was 97.03 %. One hundred fifteen SLNs were excised by CEUS, which was less than blue dye. The positive rates of SLNs identified by CEUS was 35.7 % (41/115), significantly higher than that by blue dye (21.8 %, 46/211). Rates of axillary seroma and infection in patients who had five or more SLNs removed are significantly higher than those with up to four nodes removed [29]. The characteristic advantage of CEUS and microbubbles is the real-time imaging for SLNs. A dynamic process of SLN enhancement from the afferent lymphatic vessel to efferent vessel is visible, and the very first developed lymph nodes are easily identified and located with a guidewire. Therefore, the number of SLNs detected by CEUS was less than that detected by blue dye in our study, but there was no significant difference between the identification rates by CEUS and blue dye. In other words, maybe SLNB with CEUS can adequately stage the axilla with fewer traumas. The false negative rate of SLNB is the most important concern of the surgeons. It was reported that SLNB had an inherent false negative rate in the range of 4–10 % [33, 34]. In our study, all the axillary positive cases were diagnosed correctly by CEUS except for three undeveloped cases, which underwent ALND consequently and were proved axillary positive pathologically. Therefore, no patient delayed her treatment. There was no unnecessary ALND in the consequence of CEUS, either. However, one case was misdiagnosed negative by blue dye, which may lose the opportunity of ALND without CEUS.

The use of microbubbles and CEUS may also be of value in the diagnosis of involved SLN. The normal direction of internal drainage of lymph nodes is from cortex to cortex, which appeared to be enhanced from peripheral region to central zone in CEUS. SLNs that contained US-detectable metastases demonstrated areas did not enhance as a result of tumor displacement or destruction of the normal tissue. Goldberg had reported that the sensitivity and specificity of CEUS in predicting SLNs metastases in a swine model were 95 and 63 %, respectively, with an overall accuracy of 86 % [31]. In another 20-case study, inhomogeneous enhancement was also observed in 2 metastasized cases. Peripherally increased echogenicity or longer enhancing period was observed because of the replacement of the inner portion of the LN by metastatic lesions [32]. However, there are few reports about the value of the CEUS in predicting lymph node metastases. In our study, we summarized the enhancement pattern into three types and obtained an accuracy rate of 85.2 %, compared with the pathology result. Although the result is not very satisfactory, it is successfully correlated with the intra-nodal filling defects with tumor involved. This may be a valuable addition to the technique.

## Conclusions

Microbubbles and CEUS is a feasible approach for SLN identification. The enhancing patterns on CEUS may be helpful to recognize the metastasizing SLNs. This novel method may be a promising technique for the clinical application. Larger patient data and multicenter cohort trials are required and long-term follow-up data on the safety and therapeutic effect are still needed.

### Ethics approval and consent to participate

The study was approved by the institutional ethics committee of Peking University People’s Hospital (IRB approval number is 2012045). Informed consent was obtained from all enrolled patients under the ‘Ethics, consent and permissions’ heading (The version number is modified revision 2012.12.18).

### Consent for publication

Informed consent for publishing the individual patient data was obtained from all the participants.
